# Exploring transnational LGBTQI+ solidarities: A scoping review protocol on the complexities, theories, and models

**DOI:** 10.1371/journal.pone.0333219

**Published:** 2025-12-29

**Authors:** Reem Alameddine, Ahmed Hamila, Edward Ou Jin Lee

**Affiliations:** 1 Université du Québec à Montréal, Montréal, Canada; 2 Université de Montréal, Montréal, Canada; Public Library of Science, UNITED KINGDOM OF GREAT BRITAIN AND NORTHERN IRELAND

## Abstract

On August 28, 2022, the Government of Canada unveiled its first Federal Action Plan to promote LGBTQI+ equality, committing to significant international investments and diplomatic initiatives. While these initiatives are viewed positively by some experts, others critique them as manifestations of homonationalism or homocolonialism, perpetuating colonial legacies and neoliberal norms. These debates underscore the complexities of transnational LGBTQI+ solidarity, particularly within the Majority World, shaped by colonial histories. This scoping review protocol aims to critically assess the empirical literature on transnational LGBTQI+ solidarities, to map, compare, and contrast various configurations of LGBTQI+ transnational solidarities, while also highlighting the different theories and methodologies employed in research on these topics. We will systematically search social science databases for relevant studies, emphasizing literature from peer-reviewed journals. In the study selection process, we will include research using primary sources and systematic reviews, while excluding the grey literature and non-peer-reviewed sources. Data will be charted using the COVIDENCE software, with rigorous inclusion criteria and a detailed data extraction process. The review will synthesize findings to identify key themes, gaps in knowledge, and provide policy and practice recommendations. Our goal is to map and compare different LGBTQI+ transnational solidarity strategies, contributing to a deeper understanding of these complex issues, ultimately informing future research and policy development in the field of LGBTQI+ rights on a global scale. The results will help develop a comprehensive understanding of the issues and propose a future research agenda.

## Introduction

On August 28, 2022, the Government of Canada unveiled its very first Federal Action Plan to promote the equality of Two-Spirit, lesbian, gay, bisexual, trans, queer, intersex, etc., (2SLGBTQI+) people. While this action plan focuses primarily on the Canadian context, several measures specifically target the advancement of LGBTQI+ rights internationally. Thus, the Canadian government commits “through Canada’s Feminist International Assistance Policy designed by Global Affairs Canada [to] continue investing in 2SLGBTQI+ community projects and [to] allocate up to 10 million dollars per year to the advancement of human rights as well as the socioeconomic improvement of 2SLGBTQI+ individuals in developing countries starting from 2025-2026” [1 p. 25, free translation]. In another measure, the action plan commits to “leading initiatives to renew the protection and promotion of 2SLGBTQI+ community rights globally through diplomacy and advocacy at multilateral, regional, bilateral, and international levels” [1 p. 25, free translation].

The Canadian government is not the only governing entity to engage in such initiatives. Only a few months before Canada, the European Union also launched its first-ever LGBTQI+ Equality Action Plan. Similar to the Canadian case, the European Union committed itself internationally to “set an example, showing solidarity and strengthening resilience to protect and advance the rights of LGBTQI+ people around the world, and to contribute to a global recovery that enables everyone to thrive socially and economically and leaves no one behind” [2 p. 19, free translation]. To this end, the European Union pledged to support LGBTQI+ equality actions through the Neighbourhood, Development and International Cooperation Instrument (NDICI), the Instrument for Pre-accession Assistance (IPA), and the Asylum and Migration Fund.

While Canada promotes LGBTQI+ rights on the international stage, its domestic policies have often been less progressive, revealing contradictions between its external commitments and internal realities [3]. Furthermore, in the Canadian context, it is challenging to fully understand what transnational solidarities entail and how they are practically expressed [4]. Yet, some specialists view these commitments as a positive evolution, since the promotion of LGBTQI+ rights “is indeed a good and necessary step in promoting human rights for the whole population, for global public health, and for broad development objectives, including poverty mitigation and maternal health” [5 p. 72]. However, others are skeptical of these bursts of transnational solidarity, seeing them instead as manifestations of homonationalism [6], or homocolonialism [7], which reinforces a very narrow form of sexual democratization [8], a process by which Northern countries define, celebrate, and control a normative and supposedly immutable set of sexual and gender identity categories [9,10]. Thus, far from being liberating, some of these transnational solidarity initiatives instead perpetuate structural power and privileges inherited from colonization and thus contribute to the imposition of neoliberal norms on local cultures and autonomies [11,12].

These issues surrounding transnational solidarity and LGBTQI+ rights are particularly acute in certain Majority World contexts. While the laws that criminalize same-gender relationships in several postcolonial countries often originate from colonial-era legal frameworks imposed by European powers such as France and Great Britain [13], contemporary forms of homophobia also reflect complex intersections of local cultural, religious, and political dynamics. Recognizing both colonial legacies and current sociopolitical contexts is essential to understanding the diverse realities shaping LGBTQI+ experiences across Africa and Asia. Furthermore, one of the main challenges lies in the asymmetries of influence and access between actors from the Minority World and Majority World, where funding, expertise, and discursive frameworks are often shaped by institutions and organizations historically rooted in Europe and North America [14]. Therefore, it is necessary to examine this heritage and how it articulates in the current context characterized by transnational solidarity initiatives supported and/or led by governmental entities around LGBTQI+ issues. To achieve this objective and document the state of knowledge on forms of LGBTQI+ transnational solidarities and their inherent limits, a scoping review will be conducted. We believe that conducting this scoping review will allow us to map, compare, and contrast the different models of existing LGBTQI+ transnational solidarities on a global scale, as well as highlight the various theories and methodologies used in research on these issues.

## Methods

This scoping review protocol was developed in accordance with the PRISMA-ScR (PRISMA Extension for Scoping Reviews) guidelines [15]. For our scoping review, we will follow Peter et al.’s nine-stage methodological framework [16], which includes: 1) defining the research question; 2) defining inclusion and exclusion criteria; 3) developing a research plan; 4) searching for evidence; 5) selecting relevant studies; 6) extracting the data; 7) analyzing and synthesizing the data; 8) presentation of the results; 9) summarizing the evidence, making conclusions and noting any implications of the findings. This planning process is iterative and will allow researchers to adopt a reflective approach at each stage to ensure the comprehensiveness of the work [16]. This plan will be followed throughout the scoping review process and will be documented at each stage to ensure transparency and consistency.

### Stage 1: Identifying the research question

The purpose of this scoping review is to critically assess peer-reviewed empirical literature on the forms of transnational LGBTQI+ solidarities and their inherent limitations. In doing so, our analysis aims to address several specific research questions arising from this objective:

1. What defines transnational LGBTQI+ solidarity, and how power inequalities between countries shape Minority-Majority World dynamics and their foundations?
2. Why do institutional and activist actors from both the Minority World and the Majority World engage in transnational LGBTQI+ solidarity, and what influences their motivations and priorities?
3. How is transnational LGBTQI+ solidarity implemented in practice, and how do power relations between Minority and Majority World countries shape its strategies and modalities?

These three research questions are deeply interconnected and collectively offer a comprehensive and multidimensional understanding of transnational LGBTQI+ solidarity. The first question lays the conceptual groundwork by interrogating how such solidarity is defined and how it is shaped by Minority–Majority World dynamics. In this review, the terms Minority World and Majority World are adopted within a decolonial framework that seeks to move beyond the hierarchical and geographically reductionist connotations of “Global North” and “Global South” [17,18]. Following existing scholarship [17,18], these terms refer to population-based and historically structured zones of economic and political power rather than strict geographical boundaries. However, we use them here heuristically to emphasize power relations and global asymmetries rather than to denote fixed or homogeneous regions. We also acknowledge the limitations of this binary framing, as it does not fully capture the complexity of Majority World–Majority World or Minority World–Minority World solidarities, which play equally important roles in contemporary transnational activism. The second question builds on this by exploring the motivations and priorities of the diverse actors involved—both institutional and activist—shedding light on the strategic and political considerations that guide their engagement. The third question turns to the practical implementation of solidarity, focusing on how power relations influence its forms and practices. Together, these questions allow for a critical examination of transnational LGBTQI+ solidarity as a contested and evolving field shaped by asymmetries, negotiations, and collective aspirations.

To ensure a better understanding of the research questions in this scoping review, we believe it is necessary to provide definitions for some key concepts. Firstly, we define LGBTQI+ as any one or more of the following: lesbian, gay, bisexual, transgender, queer, and intersex [19]. The term Two-Spirit (TS) will explicitly not be included when we refer to LGBTQI+ transnational solidarity initiatives because this term originates and is used by LGBTQI+ and Two-Spirit Indigenous Peoples specifically within the North American context. SOGI is an acronym we will use in keyword searches for sexual orientation and gender identity. The term “Majority World” refers to the regions and societies where most of the world’s population lives. It encompasses diverse social, political, and economic contexts that have historically been described through reductive categories such as “developing” or “Global South” [17,18]. The expression emphasizes demographic and cultural plurality and invites recognition of the wide range of knowledge systems, histories, and lived experiences that exist across these regions [17,18]. The term “Minority World’ designates the regions where a smaller proportion of the global population resides, often referred to in traditional literature as the “developed” or “Global North” [17,18]. This terminology shifts the focus away from presumed economic or political dominance, instead underscoring demographic proportion, epistemic diversity, and the interdependence that connects all regions of the world [17,18]. Transnational solidarity refers to the collaborative and reciprocal efforts of individuals, groups, and organizations that transcend national borders to pursue shared goals of justice, equity, and human rights [20]. Rather than assuming a one-directional flow of support or influence between regions, this concept recognizes the multiplicity of relationships, exchanges, and mutual learning processes that constitute global solidarities in practice [20]. It often emerges in response to issues such as human rights, environmental justice, and social inequalities, and is characterized by mutual support, shared resources, and coordinated strategies to address challenges affecting multiple communities across the globe [20].

### Stage 2: Identifying relevant studies

A meeting will be scheduled with the departmental librarian at the Université de Montréal to develop a research strategy. This meeting will help identify the main social science databases in both French and English. Keywords will be systematically searched in the identified databases and will be divided into four categories as follows:

- Category of keywords related to sexuality and LGBTQI+ issues: sexuality, LGBTQI + , SOGIE, SOGI.
- Category of keywords related to solidarity issues: solidarity, activism, advocacy, human rights, cooperation, social movements, mobilization.
- Category of keywords related to the decolonial approach: decolonial, anti-colonial, postcolonial, neocolonial, critical, North/South, Majority/Minority World.
- Category of keywords related to the transnational scale: transnational, international, global.

### Stage 3: Study selection

As part of this scoping review, we will focus on synthesizing the empirical literature relevant to our research topic. By “scientific literature”, we mean research using primary sources, scoping reviews, systematic reviews, meta-syntheses, textual analysis (media, public policy, legal), case law studies, historical archive research, and auto-ethnography. All publications included in this scoping review must come from peer-reviewed scientific journals. The time frame for the included literature will be 10 years, from 2014 to 2024, reflects the significant evolution of transnational LGBTQI+ solidarity initiatives and related issues over the past decade. This period has been particularly marked by emergence of new forms of activism, policy changes, and cross-border collaborations, both in Canada and internationally. To ensure a rigorous selection of studies, the established inclusion criteria are as follows: 1) research using primary sources employing qualitative, quantitative, or mixed research methods; 2) systematic reviews, if they contain meta-syntheses or meta-analyses; 3) theoretical articles, literature reviews, annotated bibliographies, and discussion papers; 4) conference papers (if published in scientific journals); literature written in English or French. Although the inclusion of systematic reviews in scoping reviews remains debated, we chose to include those that contain meta-syntheses or meta-analyses, as they produce new and integrated knowledge rather than merely summarizing existing studies [21]. Such reviews provide analytical interpretations that align with our objective of mapping theoretical, methodological, and empirical developments in the field.

To maintain methodological consistency, grey literature and research results or narratives found on conventional or alternative media sites (e.g., websites, newspapers, etc.) will be excluded. Additionally, doctoral theses, master’s dissertations, book chapters, books, case law, legislation, and public policies will also be excluded.

### Stage 4: Charting the data

All identified literature will be imported into COVIDENCE, the software chosen for this scoping review. This software will allow the research team to keep track of the scoping review process (up until the data extraction phase). All knowledge sources included will be reviewed by a minimum of 2 research team members. Initially, during the selection process, all duplicates will be removed, and relevant sources will be retrieved. Sources that do not meet the inclusion criteria will be excluded. In cases where there is a disagreement between the two team members regarding a source, a third evaluator will decide on its relevance and make the final determination, at which stage the reasons for exclusion will be documented. The data extraction will be completed by 2 research team members for each knowledge source. The entire process of source inclusion will be presented in a PRISMA Flow diagram [22]. In accordance with the Joanna Briggs Institute (JBI) guidance and the PRISMA-ScR framework, this scoping review does not include a formal quality assessment of the selected studies, as the objective is to map the range and nature of existing evidence rather than to evaluate methodological rigor [21].

The data to be extracted and tabulated will include study title, authors, type of empirical knowledge source, country of origin, year of publication, study objectives, theoretical framework, methodological approach, duration of study, data collection tools, recruitment strategy, participant sample, participant numbers, key inclusion or exclusion criteria. Additionally, the summary of main findings will cover key findings, key recommendations related to policy and practice implications, identified knowledge gaps and study limitations.

### Stage 5: Collating, summarizing and reporting the results

Analysis and synthesis will be undertaken by the whole team. Group meetings at points throughout to ensure consistency against original research questions, aims and objectives. Using the diagram outlining our source selection methodology and detailed tables presenting the results of these sources, we will also provide a descriptive synthesis of the findings. The synthesis will follow a descriptive and thematic approach, consistent with the methodological framework proposed by Peters et al. [16]. This process will involve coding the extracted data, identifying recurrent themes, and mapping relationships between concepts, contexts, and theoretical perspectives [16]. The aim is to produce an analytical narrative that highlights convergences, divergences, and gaps across studies. Indeed, we will produce a synthesis of the research results, including an identification of key themes and emerging tensions, with the aim of highlighting gaps in knowledge on the subject, providing recommendations, and proposing a future research agenda. The documents that will be used to complete the analysis (i.e., data extraction form, etc.) will be stored in the Université de Montréal secure server, managed by the principal investigator, Ahmed Hamila, and will be made available upon request, with the exception of the data processed within the COVIDENCE software. The review will also be registered with PROSPERO to enhance transparency and replicability of the methodological process.

The timeline for the project is as follows: We aim to identify the articles by early 2026. During the winter of 2026, we will conduct the coding phases and analysis. The synthesis and writing stages are planned for the summer of 2026, by which time we expect the project to be completed.

## Conclusion

The examination of recent commitments by Canada in favor of LGBTQI+ equality on a global scale highlight diverse perspectives among LGBTQI+ issues specialists. While some view these initiatives as essential progress for LGBTQI+ rights [23], others raise concerns about the potential perpetuation of neoliberal norms, homonationalism, or homocolonialism [24,25]. These debates thus reveal the complexity of surrounding transnational solidarity in the field of LGBTQI+ rights [26]. This proposed scoping review will map and compare various models of transnational LGBTQI+ solidarities, while exploring the underlying theories and methodologies. By documenting initiatives in transnational LGBTQI+ solidarity found in empirical literature, our scoping review aims to deepen our understanding of the issues related to LGBTQI+ rights on a global scale.

However, this study has certain limitations. The decision to include only studies published in English and French may exclude relevant perspectives from other linguistic contexts. Additionally, by focusing exclusively on peer-reviewed academic literature and excluding grey literature, this review prioritizes sources that meet established methodological standards by may overlook valuable insights from reports, policy documents, and activist-produced knowledge. Moreover, the rapid evolution of the field means that more recent or emerging forms of LGBTQI+ solidarity initiatives may not yet be well documented in academic literature. These choices were made to unsure feasibility and methodological consistency, yet they necessarily shape the scope of our analysis.
